# Anesthesia management for tracheoesophageal fistula closed with a new gastrointestinal occluder device: a case report

**DOI:** 10.1186/s13019-022-02038-8

**Published:** 2022-11-16

**Authors:** He Huang, Jigang Zhang, Lurong Li, Guoxin Zhang, Dechong Zhu

**Affiliations:** 1 Department of Anesthesiology and Perioperative Medicine, First Affiliated Hospital of Nanjing Medical University, Guangzhou Road 300, Nanjing, 210029 Jiangsu Province People’s Republic of China; 2 Department of Gastroenterology, First Affiliated Hospital of Nanjing Medical University, Nanjing, People’s Republic of China

**Keywords:** Esophageal cancer, Tracheoesophageal fistula, New gastrointestinal occluder device, General anesthesia, Airway management

## Abstract

**Background:**

Tracheoesophageal fistula (TEF) is a rare but life-threatening complication after esophagectomy. A new gastrointestinal occluder device provides treatment for TEF patients. However, TEF-related pneumonia and respiratory failure increase the difficulty of anesthesia management, especially in airway management.

**Case presentation:**

A 64-year-old man with thoracic esophageal cancer underwent esophagectomy and gastric tube reconstruction one year ago. The patient presented with recurrent cough and sputum after surgery. Gastroscopy revealed a fistula between the esophagogastric anastomotic site and membrane of the trachea. Therefore, the patient received implantation of a new gastrointestinal occluder device under gastroscopy combined with tracheoscopy. Airway management under general anesthesia was discussed with an interdisciplinary decision, and cuffed endotracheal tube with an inner diameter of 5.5 mm was chosen. This airway management ensured adequate oxygenation during the operation and provided sufficient space for the operation of the tracheoscope in the trachea. Finally, the TEF disappeared after the operation, and the patient was administered an oral diet on the first postoperative day.

**Conclusions:**

The implantation of a new gastrointestinal occluder device under gastroscopy combined with tracheoscopy provides a new treatment for TEF patients. This case report suggests that it is important to select an endotracheal tube with an appropriate inner diameter that can not only meet the requirements of ventilation but also does not affect the operation of tracheoscopy in the trachea.

**Supplementary Information:**

The online version contains supplementary material available at 10.1186/s13019-022-02038-8.

## Background

Tracheoesophageal fistula (TEF) is a life-threatening complication after esophagectomy, although its incidence is approximately 0.3% [1]. TEF-related pneumonia and respiratory failure significantly reduce postoperative quality of life and increase postoperative mortality [2, 3].

Although the muscular flap, pericardiac flap or thymus flap has been used for TEF repair under the thoracoscopic approach [4, 5], most TEF patients are inoperable. Currently, the endoscopic repair of TEF, such as self-expanding metal stents, degradable stents, silicone stents and endobronchial one-way umbrella-shaped valves, has been used to cover the fistula and relieve patient pain [6, 7]. However, the endoscopic repair of TEF brings several challenges for anesthesia management. In this case, one patient who suffered TEF after esophagectomy was successfully cured with the implantation of a new gastrointestinal occluder device under gastroscopy combined with tracheoscopy. Here, we report the anesthesia management, especially the airway management, of this patient.

## Case presentation

A 64-year-old male underwent esophagectomy one year ago, and the patient revealed recurrent cough, sputum and choke after eating. A thoracic computed tomography scan showed that the location of the fistula was at the level of the second thoracic vertebra. The preoperative multidisciplinary consultation concluded that the patient was not suitable for thoracoscopic surgery due to the cachexia state, and implantation of a new gastrointestinal occluder device under gastroscopy combined with tracheoscopy was an appropriate treatment. To ensure that the fiberoptic bronchoscope and the endotracheal tube could be placed in the trachea in parallel, cuffed endotracheal tube with an inner diameter of 5.5 mm was selected after interdisciplinary discussion, which ensured adequate oxygenation during the operation and provided sufficient space for the operation of the fiberoptic bronchoscope in the trachea.

To avoid aspiration resulting from esophageal reflux due to positive pressure ventilation of the mask during induction, gastroscopy was performed before anesthesia to ensure preoperative gastric emptying. The patient was monitored with electrocardiogram, pulse oxygen saturation and invasive arterial blood pressure. After adequately preoxygenation, anesthesia was induced by rapid sequential induction of rocuronium 0.6 mg/kg, etomidate 0.3 mg/kg, midazolam 0.02 mg/kg and fentanyl 5 μg/kg. Under the guidance of a fiberoptic bronchoscope, the end of the endotracheal tube was precisely placed at the distal end of the fistula, which could prevent gastric distension during positive pressure ventilation. Anesthesia was maintained with propofol 4–8 mg/kg/h and remifentanil 0.05–0.1 μg/kg/min to a target bispectral index of 45–55. Mechanical ventilation was performed in the volume control mode after intubation, with tidal volume 6–8 ml/kg, respiration rate 12–14 times/min, and fraction of inspiration O_2_ (F_i_O_2_) 60%.

**Fig. 1:**
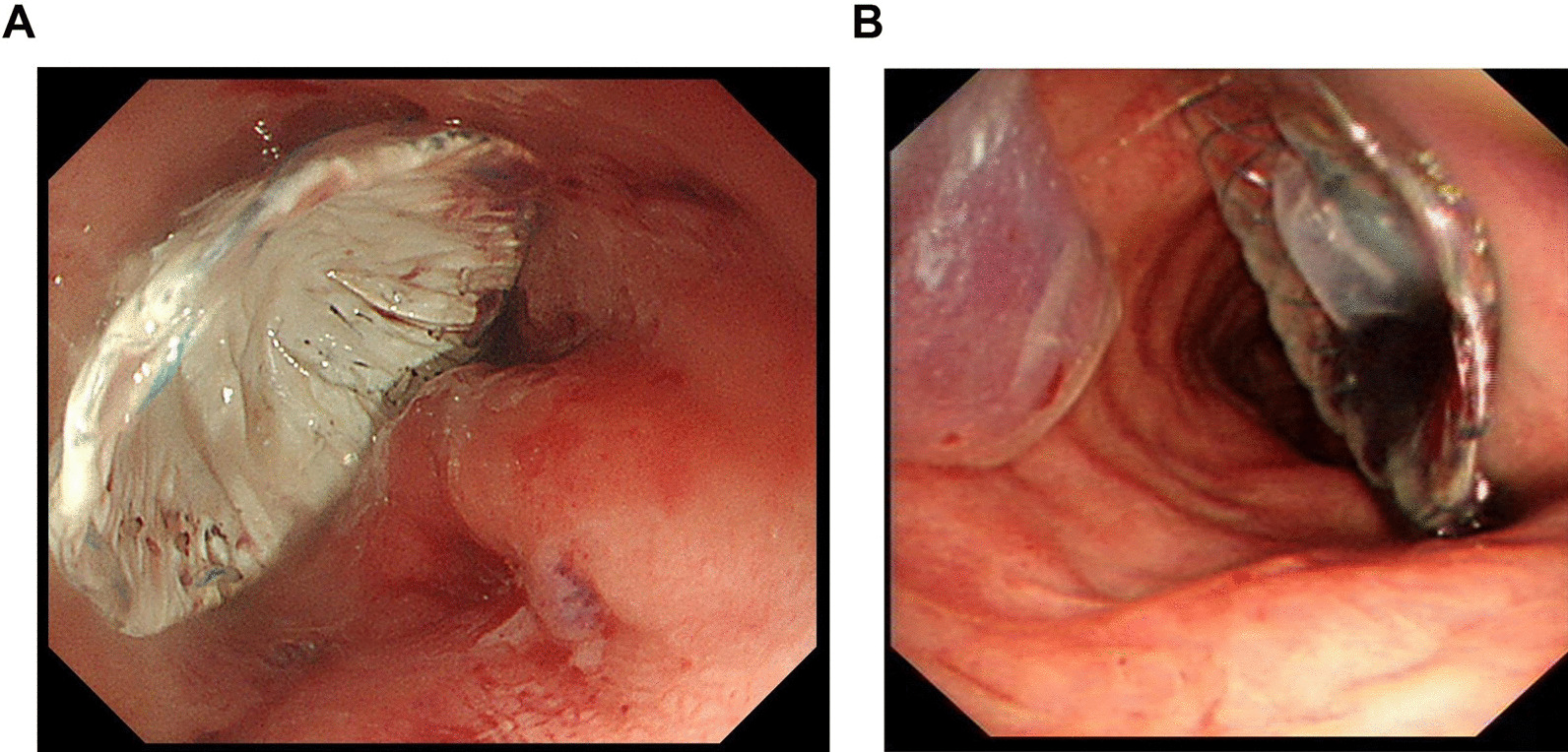
**A** Esophageal views of the occluder device. **B** Fiberoptic bronchoscope views of the occluder device

At 20 cm away from the incisors, gastroscopy revealed a fistula approximately 1 cm in diameter between the esophagogastric anastomotic site and the membrane of the trachea. Under the guidance of gastroscopy combined with tracheoscopy, endoscopic physicians completely repaired the TEF, and the esophageal side of the new gastrointestinal occluder device was correctly placed (Fig. 1A). The fiberoptic bronchoscope image showed the end of the endotracheal tube, and the tracheal side of the occluder device was also correctly placed (Fig. 1B). The hemodynamics and oxygenation were stable during the operation.

The patient had no symptoms such as speech disorders, dysphagia or cough after the implantation of the device, and administered an oral diet the day after the operation. Finally, the patient was discharged from the hospital on the second day after operation.

## Discussion and conclusions

Patients with TEF usually have severe pulmonary infection, malnutrition and other conditions, and primary or recurrent TEF is an important challenge for airway management. At present, minimally invasive thoracoscopic approaches or thoracostomy are important methods for treating TEF, but some patients cannot tolerate surgical stress. With the development of endoscopic repair technology, minimally invasive treatment of TEF has achieved great progress. In the past ten years, cases of congenital heart disease closure devices for the treatment of TEF have been reported. Compared with the congenital heart disease closure device, the new gastrointestinal occluder device (designed by Zhang et al. [8]) is made of laminated nitinol mesh with two self-expanding discs connected by a thin waist (Additional file 1: Fig. S1A, B). The occluder device has a lighter weight on both sides of the umbrella disc, which can reduce the pressure on the fistula tissue to reduce the risk of necrosis, bleeding, displacement, shedding, and even suffocation.

At present, there is no unified standard anesthesia management for the repair of TEF under endoscopy. The monitored anesthesia care without intubation requires a relatively small dose of anesthetic; however, the depth of anesthesia may not meet the requirements of the endoscopy operation, and the risks of hypoxemia and aspiration resulting from esophageal reflux are significantly increased intraoperatively. Most TEF patients are complicated with long-term pneumonia, which increases the difficulty of extubation after general anesthesia, but the establishment of an artificial airway can ensure the stability of intraoperative oxygenation. After a preoperative multidisciplinary consultation, endotracheal intubation to control ventilation was relatively safe for this patient.

Previous studies have demonstrated that positioning the endotracheal tube with its end lying distal to the fistula is essential for avoiding gastric distension [9]. However, the fiberoptic bronchoscope could not observe the tracheal side of the fistula when entering from the endotracheal tube. In this case, implantation of the new gastrointestinal occluder device requires the guidance of gastroscopy combined with tracheoscopy. To observe the tracheal side of the TEF, the fiberoptic bronchoscope should enter the trachea from outside the endotracheal tube. Therefore, the endotracheal tube needs to be carefully evaluated for an appropriate inner diameter to ensure that the fiberoptic bronchoscope and the endotracheal tube can be placed in the trachea in parallel.

The choice of endotracheal tube diameter in the endoscopic treatment of TEF has no unified standard to date. Tracheal anatomy shows that the normal diameter of the trachea is 1.2–1.5 cm for males and 1.0–1.2 cm for females. Considering that the fiberoptic bronchoscope diameter was approximately 0.5–0.6 cm, an endotracheal tube with an inner diameter of 5.5 mm was selected to ensure there was adequate space for the fiberoptic bronchoscope to observe the tracheal side of the TEF from outside the endotracheal tube and to guide the accurate implantation of the occluder device.

In conclusion, the implantation of a new gastrointestinal occluder device under gastroscopy combined with tracheoscopy could be a promising option for TEF repair. This case may provide a kind of airway management for implantation of the new gastrointestinal occluder device; however, the efficacy and safety of this protocol needs to be verified in more cases.

## Supplementary Information

**Additional file 1: Fig. S1.** The new gastrointestinal occluder device. **A** Vertical view. **B** Lateral view.

## Data Availability

All data analyzed during this study are included in this published article.

## Abbreviations

TEF: Tracheoesophageal fistula
F_i_O_2_: Fraction of inspiration O_2_
