# Supplementary figures and images for: Anesthesia management for tracheoesophageal fistula closed with a new gastrointestinal occluder device: a case report
Source: J Cardiothorac Surg. 2022 Nov 16;17:287. doi: 10.1186/s13019-022-02038-8 (PMC9670478; doi:10.1186/s13019-022-02038-8)

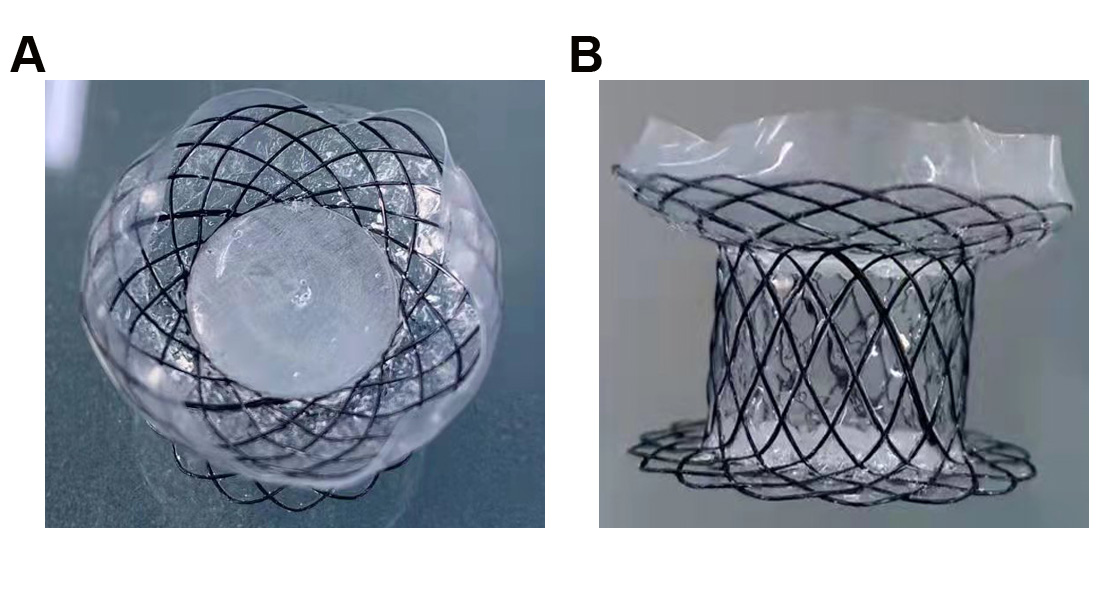

Supplement: Supplementary file 1 — Additional file 1: Fig. S1. The new gastrointestinal occluder device. A Vertical view. B Lateral view. [file 13019_2022_2038_MOESM1_ESM.jpg]
